# Volunteer Bias in Recruitment, Retention, and Blood Sample Donation in a Randomised Controlled Trial Involving Mothers and Their Children at Six Months and Two Years: A Longitudinal Analysis

**DOI:** 10.1371/journal.pone.0067912

**Published:** 2013-07-09

**Authors:** Sue Jordan, Alan Watkins, Mel Storey, Steven J. Allen, Caroline J. Brooks, Iveta Garaiova, Martin L. Heaven, Ruth Jones, Sue F. Plummer, Ian T. Russell, Catherine A. Thornton, Gareth Morgan

**Affiliations:** 1 Department of Nursing, The College of Human and Health Sciences, Swansea University, Singleton Park, Swansea, Wales, United Kingdom; 2 The College of Medicine, Swansea University, Singleton Park, Swansea, Wales, UK Wales, United Kingdom; 3 Obsidian Research Limited, Baglan Industrial Park, Port Talbot, West Glamorgan, Wales, United Kingdom; Cardiff University, United Kingdom

## Abstract

**Background:**

The vulnerability of clinical trials to volunteer bias is under-reported. Volunteer bias is systematic error due to differences between those who choose to participate in studies and those who do not.

**Methods and Results:**

This paper extends the applications of the concept of volunteer bias by using data from a trial of probiotic supplementation for childhood atopy in healthy dyads to explore 1) differences between a) trial participants and aggregated data from publicly available databases b) participants and non-participants as the trial progressed 2) impact on trial findings of weighting data according to deprivation (Townsend) fifths in the sample and target populations. 1) a) Recruits (n = 454) were less deprived than the target population, matched for area of residence and delivery dates (n = 6,893) (mean [SD] deprivation scores 0.09[4.21] and 0.79[4.08], t = 3.44, df = 511, p<0.001). b) i)As the trial progressed, representation of the most deprived decreased. These participants and smokers were less likely to be retained at 6 months (n = 430[95%]) (OR 0.29,0.13–0.67 and 0.20,0.09–0.46), and 2 years (n = 380[84%]) (aOR 0.68,0.50–0.93 and 0.55,0.28–1.09), and consent to infant blood sample donation (n = 220[48%]) (aOR 0.72,0.57–0.92 and 0.43,0.22–0.83). ii)Mothers interested in probiotics or research or reporting infants’ adverse events or rashes were more likely to attend research clinics and consent to skin-prick testing. Mothers participating to help children were more likely to consent to infant blood sample donation. 2) In one trial outcome, atopic eczema, the intervention had a positive effect only in the over-represented, least deprived group. Here, data weighting attenuated risk reduction from 6.9%(0.9–13.1%) to 4.6%(−1.4–+10.5%), and OR from 0.40(0.18–0.91) to 0.56(0.26–1.21). Other findings were unchanged.

**Conclusions:**

Potential for volunteer bias intensified during the trial, due to non-participation of the most deprived and smokers. However, these were not the only predictors of non-participation. Data weighting quantified volunteer bias and modified one important trial outcome.

**Trial Registration:**

This randomised, double blind, parallel group, placebo controlled trial is registered with the International Standard Randomised Controlled Trials Register, Number (ISRCTN) 26287422. Registered title: Probiotics in the prevention of atopy in infants and children.

## Introduction

Recruitment to trials is deteriorating, particularly in developed countries [1]: industry sources suggest that recruitment rates across all trials fell by 75% between 1999–2002 and 2003–2006 [2]. Similarly, the proportion of recruits withdrawing from trials steadily increased between 1955 and 2000 [3], prompting the US Food and Drug Administration (FDA) to insist on measures to minimise missing data and the consequent bias [4].

Bias arising within trials, due to systematic differences between trial arms, threatens internal validity [5]. In addition, the external validity, generalisability, transferability and utility of well-conducted trials may be threatened where recruited and retained samples are less than 100% of the target population. Such selection bias, defined as the introduction of error due to systematic differences in the characteristics between those selected and those not selected for a given study [6], renders the recruited sample unrepresentative of the target population [7]–[9].

Recruitment of volunteers is a potential source of selection bias [10]. Where a sample can contain only those willing to participate in the study or experiment, systematic differences may arise between those who volunteer and those who decline or do not respond to invitations. Such “volunteer bias” is defined as any process at any stage of inference which tends to produce results or conclusions that differ systematically from the truth, arising where volunteers from a specified sample may exhibit exposures or outcomes which differ from those of non-volunteers [11]. Volunteer bias may arise during recruitment, retention, participation in follow-up clinics [12], and consent to blood sample donation. Volunteers may differ from the target population not only in socio-demographic characteristics, but also in less tangible ways, such as perceptions of the study’s leverage, saliency or relevance [13], or altruism [14].

The antithesis of volunteer bias, non-response bias [11], has been well scrutinised in surveys [13], [15] and observational studies requiring consent [16]. Although trials suffer higher non-response rates than surveys or observational studies [17]–[19], analysis of trial data rarely accounts for volunteer bias [7], [20]. Searches of three databases (PubMed, Web of Science, Scopus) located no reports of predictors of participation and consent to sample donation by well infants in clinical trials, using the key word/MeSH term combination: randomised controlled trials, pregnant women, infants, preventive therapy, research subject recruitment, loss to follow up, non-response bias, with or without “blood specimen collection”.

Little is known about families who decline to participate in clinical trials [21]. While there are exceptions, such as parents of seriously ill children [14], and situations where research offers the only access to free medication [22], non-targeted recruitment in all research designs favours healthier, wealthier, better educated, non-smokers, risking volunteer bias [10], [12], [17]–[19], [23]–[27]. The potential consequences of volunteer bias might be summarised [28]:

- Volunteer bias threatens the **generalisability** or external validity, transferability, and utility of findings and detracts from their clinical value [20]. When ‘hard to reach’ sections of the population are not included in a study, there can be no certainty that findings will be applicable to them. If a trial has been conducted in a population judged to be over-restricted, dissimilar or unrepresentative, findings may be dismissed as irrelevant. Prevention or vaccine trials are particularly vulnerable to such criticisms [7], [19], [26], [29]–[33].
- The incidence of disease in the recruited sample may be lower than accounted for in sample size calculations based on the incidence of disease in the whole population. This could leave the trial **under-powered** even when the target sample size has been recruited.
- Where trials report on conditions whose prevalence varies across the socio-demographic spectrum, **findings**, particularly estimates of the absolute effects of interventions (such as numbers needed to treat or harm, and costs), are often affected by over- or under-representation or exclusion of certain groups [7].

Evidence on which to base practice recommendations for wide sections of the population requires ‘Research evidence reflecting the diversity of the population’ [34], and trials with minimal demographic imbalance in recruitment and retention [28]. This paper aims to extend the application of the concept of volunteer bias to clinical trials, using data from a paediatric trial, by exploring:

1. **Potential for Volunteer Bias**

- Differences between the recruited sample and the target population.
- Impact on retention, clinic attendance, consent to skin-prick testing and blood sample donation by well infants of i) demographics ii) leverage, saliency and altruism.

1. **Adjustment for potential volunteer bias by weighting outcome data** [8] according to material deprivation (Townsend) fifths.

## Methods

### Ethics Statements

- Ethical approval was granted in February 2004 by the South West Wales Research Ethics Committee on behalf of NHS Wales (project ref. 2004.024). Women were given written information on the trial and data collection, and gave informed, signed consent at 36 weeks’ gestation.
- Data held in SAIL databases are anonymised and aggregated and have been obtained with permission of relevant Data Protection Officers, as approved by the National Research Ethics Service, Wales [47], [48].

### The Trial

As reported elsewhere [35], [36], this randomised, double-blind, placebo-controlled, parallel-group trial assessed the effects of probiotic food supplements on key immune parameters and prevention of atopy and atopic conditions (asthma, eczema and allergic rhinitis) in young children. Healthy women with normal singleton pregnancies under the care of clinicians in Abertawe Bro Morgannwg University Health Board, Wales, UK were recruited May 2005- October 2007. All participants were ambulatory, managed in primary care, and well or “free from disease” at recruitment, although many infants were at high or increased risk of developing atopic conditions. Inclusion criteria were: mother aged ≥16 years, normal singleton pregnancy, gestation at delivery >36 weeks, freely given, signed, informed consent to participate in the study. We excluded: women unable or unwilling to give informed consent, those with any serious medical condition affecting the woman or infant or the likely outcome of the pregnancy, families where a member of the infant’s sibship or household was already recruited to the study. Women were asked to take the probiotic supplement daily from recruitment at 36 weeks until delivery, and administer the supplement to their infants daily from birth to age 6 months.

### Sample Size

A sample of 308 infants (154 in each group) was sufficient to detect a 50% reduction in eczema frequency (40% to 20%) in the probiotic group [37] with 90% power and 1% significance. To demonstrate a similar proportional reduction in asthma at 5 years (20% to 10%) [38], 538 infants would have been required. We recruited 454 pregnant women within available resources.

### Recruitment Strategy

A multifaceted recruitment strategy was designed to contact the whole population of pregnant women in the catchment area (Table 1). Most (362, 79.7%) participants were recruited by one of seven fieldworkers, minimising the impact of the approach style of individual researchers. Written information indicated that the trial was focussed on prevention of eczema and asthma in infants and children, who were at either increased or normal risk of developing atopy. The risk factor considered was one or more family member already suffering from an atopic condition (asthma, eczema or allergic rhinitis).

**Table 1:** Recruitment strategies considered.

Strategy	Used	Advantages	Disadvantages	Findings
Non-targeted				
Written information distributed by a) midwives to all women attending booking clinics in primary care from 12 weeks’ gestation b) receptionists to all women attending hospital for routine 20 week ultrasound scan	Yes	Maximum coverage of population of pregnant women. (We estimate that >99% women in our area book for (free) antenatal care.) Not labour intensive.	Risks non-contact bias by failing to contact those not booking, typically the most disadvantaged. Assumes literacy. Information is not tailored to individuals’ needs, health beliefs or world views. Relies on health service staff.	Recruitment [13], [90], and retention [91] demanded a labour-intensive face to face approach. Written information alone was insufficient [92]: only 36/454 (7.9%) participants were recruited without a personal approach. These participants were less likely to emanate from deprived areas (U = 5627, Z = −2.05, p = 0.04, effect size, r = 0.09). Although written information was widely distributed, most (69%, 286) recruits did not recall receiving it.
Media: website, TV, local press and radio	Yes	Reaches a wide audience amongst the ‘less ill’, including partners and families [93], [94], who may influence women’s decision-making.	Advertising costs. Impact may be disappointing [75], and difficult to quantify.	We observed little impact. Following TV coverage, we received five telephone calls, all from women living outside the catchment area or already delivered. Two (0.5%) recruits first heard of the trial on TV and 7 (1.7%) via radio. We do not know whether the media had any more subtle effects in preparing families for researchers’ approaches.
Monetary incentives	No	The most effective strategy to improve recruitment. A ‘dose-response’ effect is suggested [95]–[97].	Not recommended for research involving children in the UK [98∶90].	We offered no inducements, and no-one mentioned ‘Getting things for free’ [90]. Rather, 90% (372/413) participants agreed that ‘research is everyone’s business’ [99]. There was general recognition that research could only happen and medical management could only improve if families were willing to join trials.
Targeted				
Personal approach in hospital antenatal clinics.	Yes	A personal approach tailors presentation of the trial to each individual’s health beliefs, world views or need for information [60], [100].	Insufficient resources to speak with all women. Labour intensive and therefore costly. Risks non-contact bias by excluding those not attending, typically the most disadvantaged.	A personal approach improved the socio-demographic representation of the recruited sample by allowing researchers to tailor presentation of the trial to each individuals’ need for information [60], [100], which resonates with leverage-saliency theories of participation and marketing techniques [74], [76], [101], [102].
Personal approach in community groups (in this study, parenting and aquanatal classes).	Yes	In the USA, involvement in community groups, at church or civic events, increased recruitment of women from ethnic minorities [103].	Some classes are poorly attended. Labour intensive, often outside office hours.	Only 20/454 (4.4%) women were recruited this way. Their deprivation scores were not significantly different from the whole sample. The effectiveness of this recruitment strategy is likely to be context specific.

### Research Clinics

When infants reached 6 months and 2 years of age, carers were invited to research clinics. Participants were informed at recruitment and reminded at invitation that separate signed, informed consent would be sought for skin-prick testing for common allergens (housemite, grass, cow’s milk, egg, cat) and, at 6 months only, for blood sample collection from the infant for immunological investigation. Interpretations of skin-prick testing were offered to carers immediately and could be used to modify exposure to common allergens. To minimise attrition, considerable efforts were made to contact participants, and where infants were unable to attend clinics, information was obtained by home visits or telephone interviews [39].

### Data Collection

Trial data were obtained from several sources:

1. Questionnaires (covering demographics, compliance, risk factors for atopy, signs and symptoms of atopic conditions, adverse events [40] and infant’s health) at: 36 weeks of pregnancy (recruitment), 6, 12, 18 weeks, 6 months, 1 and 2 years. At the 6 month contact, researchers asked five questions to elicit parents’ reasons for joining the trial, and their views on the trial. Responses to open questions were recorded for illustration.
2. Medical records: maternity and child health.
3. Biological Samples: maternal blood at 36 weeks’ gestation, infant blood from the umbilical cord and venepuncture at 6 months, placental tissue, breast milk at 2 and 6 weeks, stool samples at birth, 2, 6, 12, 18 weeks and 6 months.
4. Procedures: clinical examination and skin-prick tests for common allergens at 6 months and 2 years.

No information was available on non-respondents, so summary statistics relating to the target population were obtained for comparison [41] from all publically available sources:

1. 2001 Census [42] for occupation, ethnicity, and household status. Parents’ most recent occupations were coded and grouped in accordance with Office of National Statistics (ONS) [42]–[44];
2. Infant Feeding Survey [45] for smoking and alcohol use;
3. Welsh Health Survey [46] for asthma;
4. All-Wales health services’ electronic database (Secure Anonymised Information Linkage [SAIL] database) [47], [48] for material deprivation, as Townsend scores, ranks and fifths. Townsend scores are calculated from rates of unemployment, vehicle ownership, home ownership, and overcrowding for each geographical area of residence, using Lower Super Output Areas (LSOAs) defined by postcodes. [49]. We generated a comparator group within SAIL defined by:
  - precise geographical area of residence at birth, using LSOAs.
  - births during the recruitment period (May 2005 to November 2007).

Data were entered into IBM SPSS statistics v19 for Windows, in duplicate. Files were compared electronically (SPSS Data Entry Builder v4) and discrepancies reconciled before analysis.

### Analysis

a) The recruited sample was compared with external population data, listed above.
  - Retention, clinic attendance, consent to skin-prick testing for allergy, retention at 6 months and 2 years, and blood sample donation were explored in bivariate analyses, and, where feasible, by logistic regression [50], with variables as listed (Tables S1, S2). Regression models were built iteratively using i) socio-demographic variables ii) variables reflecting leverage, such as rashes, and reasons for joining the trial, such as altruism. Model parameters for each stage were compared. We checked for any attrition bias linked to trial arm.
Further analyses of the trial outcomes were undertaken to explore the potential impact of volunteer bias, as recommended [4]. Trial outcome data were weighted to reflect the distribution of deprivation (Townsend) fifths amongst respondents for each outcome relative to the target population matched in the SAIL database. The weighting factor for each fifth was calculated as that fifth’s proportion in the population divided by the proportion in the sample for each outcome (weighting factor = % in population/% in sample). SPSS statistics then created a new frequency variable by multiplying existing frequencies by the weighting factor. Associations between trial arm and clinical outcome were re-tested. Subgroups were used solely to explore the findings.

## Results

**Figure 1:**
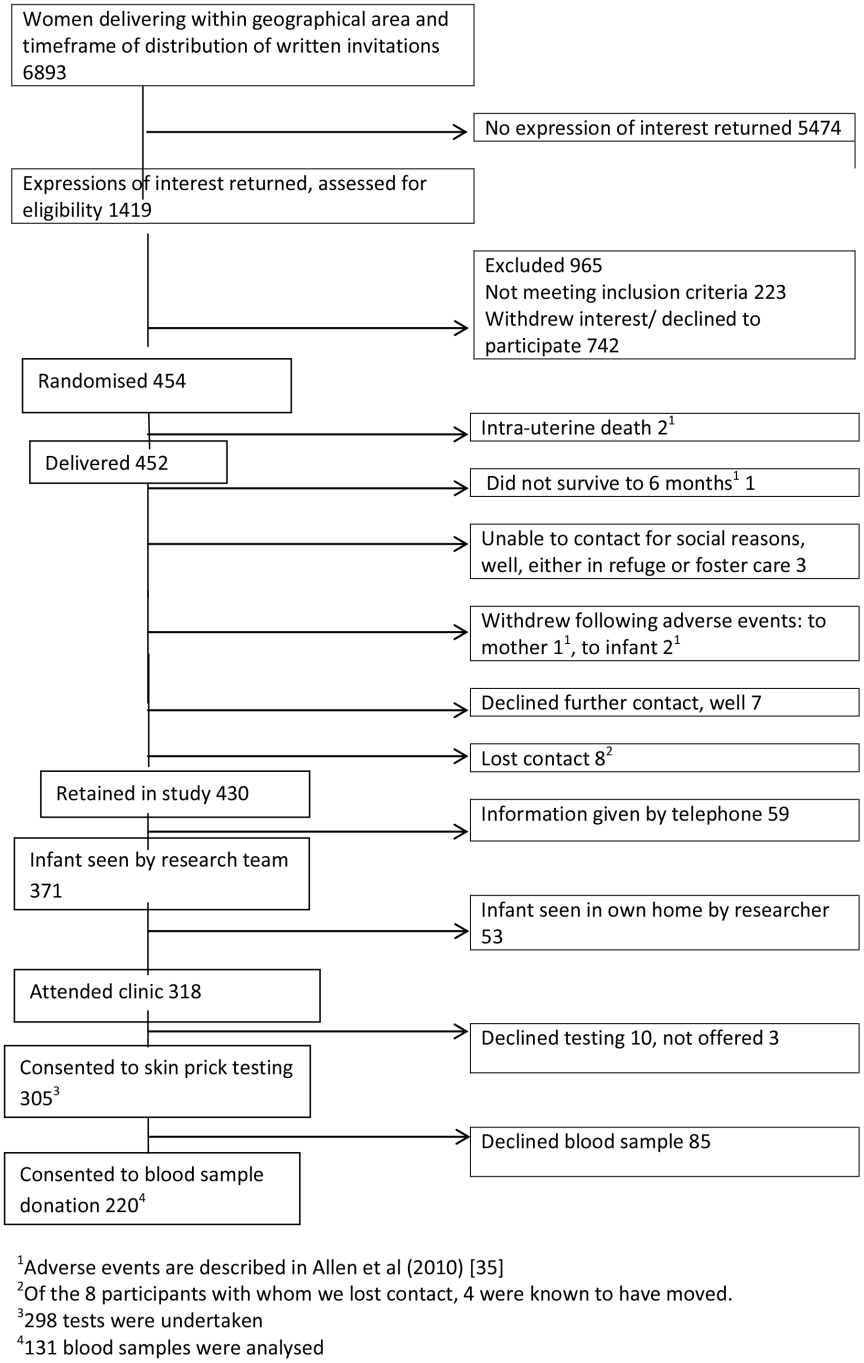
Participant Flow Diagram for observation study to 6 month contact point.

**Figure 2:**
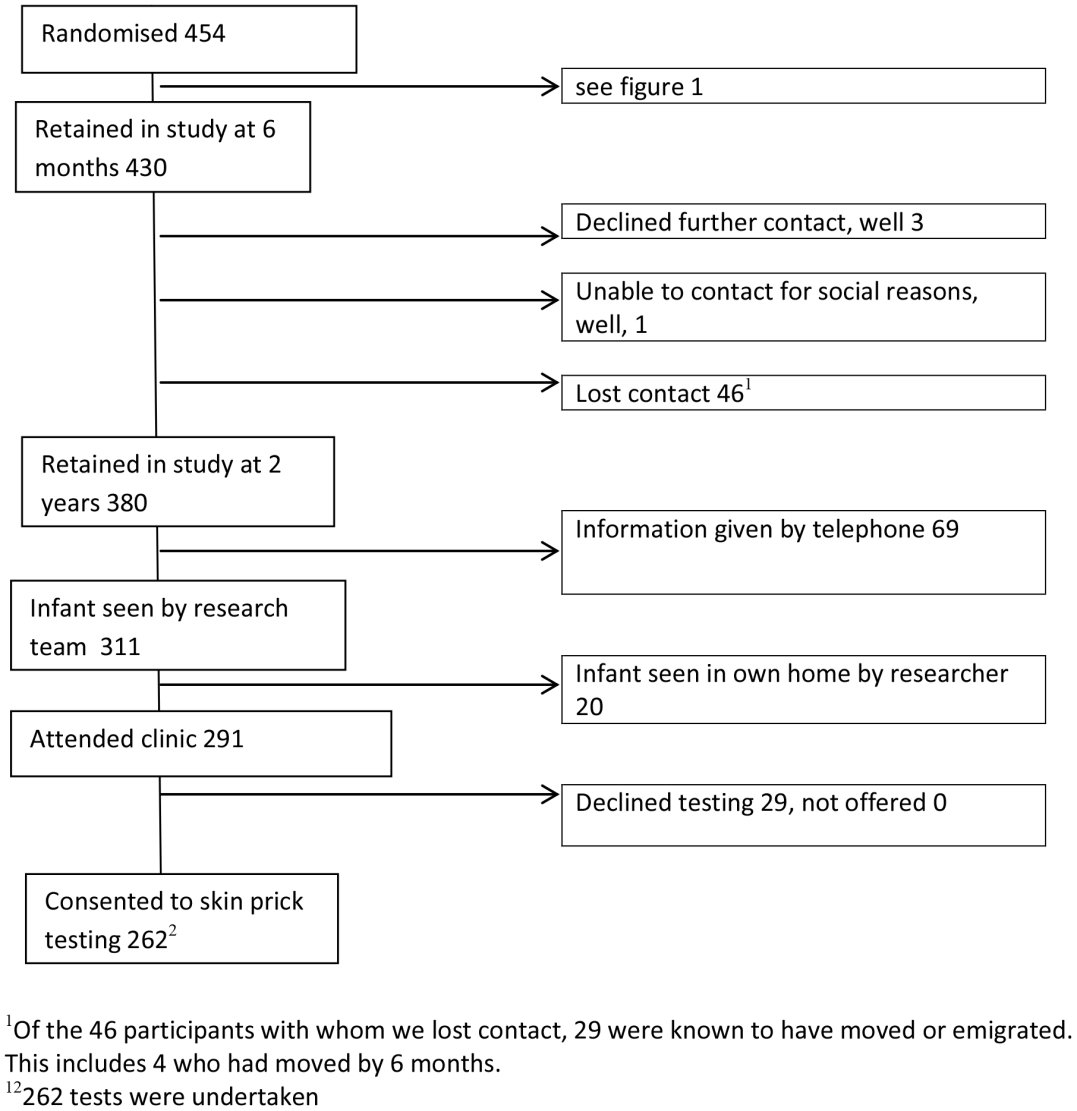
Participant Flow Diagram for observation study to 2 year contact point.

Between April 2005 and June 2007, 1419 expressions of interest were received, yielding 454 recruits (32%, 454/1419). Over the 2.25 years of recruitment, this 1419 represents almost 2% of the ∼74,000 births in Wales, and 20% of the 6,893 women delivering in the LSOAs represented in the trial as identified in the SAIL database. Attrition was 5.3% (24/454) at 6 month contact (Figure 1) and 16.3% (74/454) at 2 years (Figure 2).

### 1) Potential for Volunteer Bias

#### a) Recruitment

**Figure 3:**
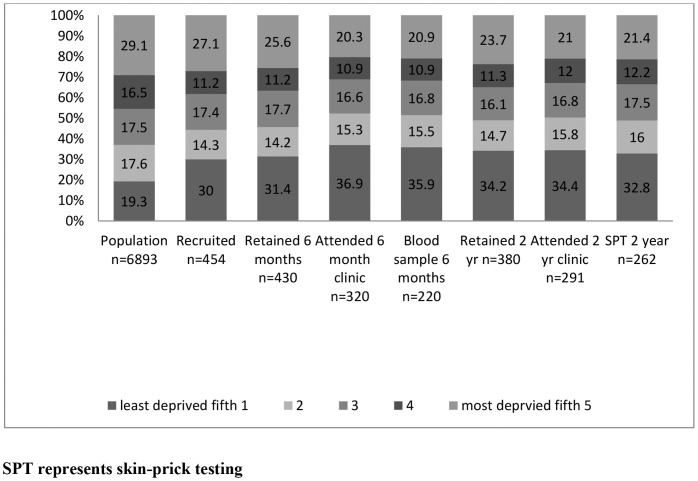
Proportion in each deprivation (Townsend) fifth in the population and each stage of the trial.

**Figure 4:**
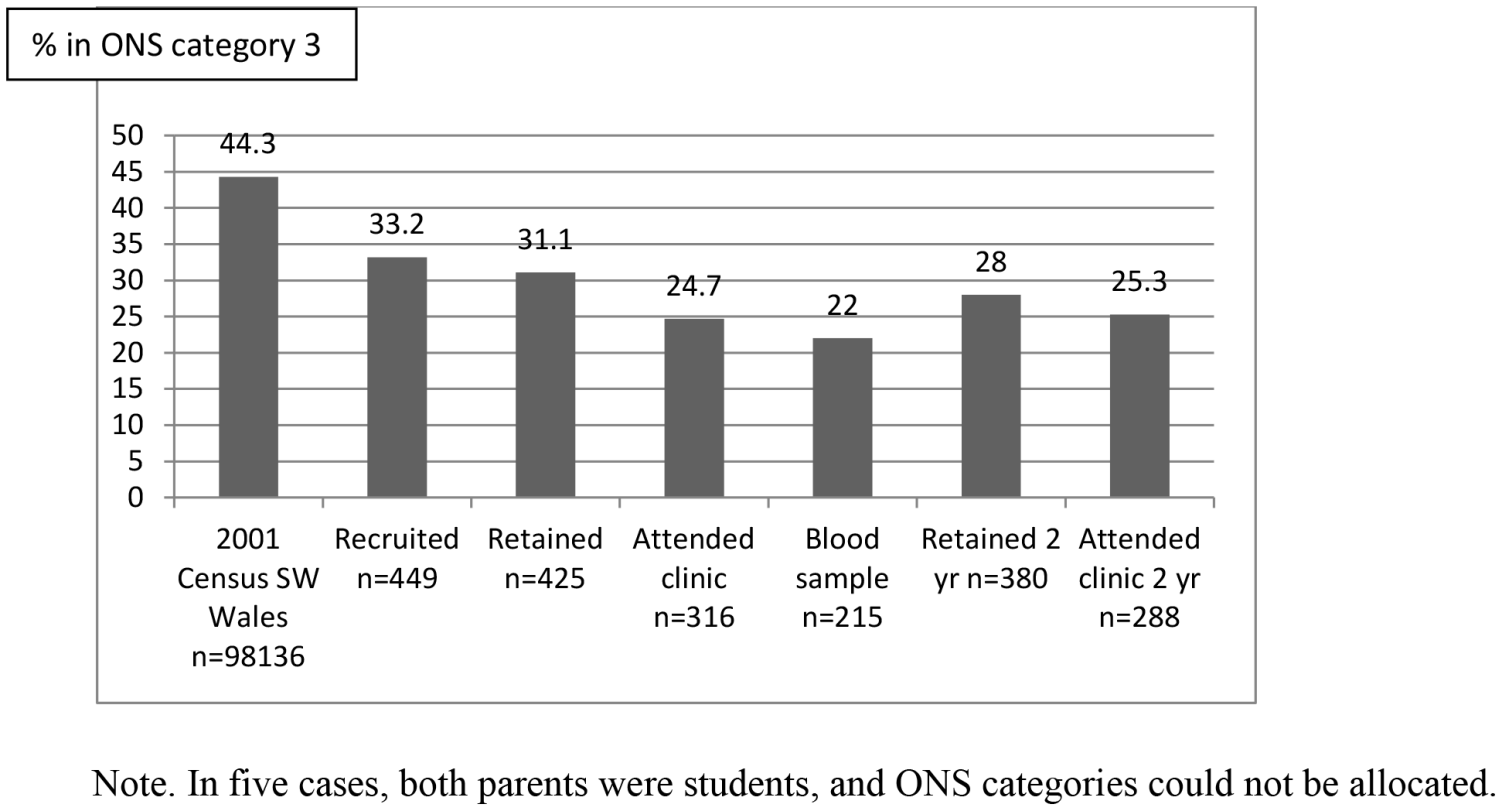
Proportion of participants from ONS Category 3 at each stage of the trial.

The recruited sample was less materially deprived than the target population closely matched for area of residence at birth (Table 2). A disproportionate number of recruits were from the least deprived (Townsend) fifth. Occupational group distributions differed between trial participants and the population of South West Wales in the 2001 Census [42] (Tables S3, S4). Both these differences intensified as the trial progressed (Figures 3, 4).

**Table 2:** Comparisons between the recruited sample and external data.

	Source of comparison data	Trial data	Comparator data	Test	
		Mean [SD]	Mean [SD]	t test^5^	significance, effect size
**Deprivation scores** 1: whole sample, 454	All-Wales health services’ electronic database, SAIL (n = 6,893)	0.09 [4.21]	0.79 [4.08]	3.44, df 511	p<0.001, r 0.15
**Deprivation rank** 1 **for Wales:** whole sample, 454	All-Wales health services’ electronic database, SAIL	925.58 [624.10]	1037.60 [591.3]	3.74, df 495	p<0.001, r 0.17
		**Number (%)**	**Number (%)**	**χ** 2 **(df 1)**	**OR, 95% CI**
**Deprivation**: least deprived fifth^1^	All-Wales health services’ electronic database, SAIL	136/454 (30%)	1,327/6,893 (19.3%)	29.9	1.79, 1.45–2.21
**Women from ONS 3** (routine occupation or never worked) 2,3	2001 Census, South West Wales [42]	149/454 (32.8%)	43,474/98,136 (44.3%)	24.14	0.61, 0.50–0.75
**Asthma as an adult: women** 2	Welsh Health Survey 2007 [46] women 16–44	104/454 (23%)	291/2,908 (10%)	61.79	2.67, 2.08–3.43
**Asthma as an adult: men** 3	Welsh Health Survey 2007 [46] Men 16–44	83/441 (19%)	203/2,541 (8%)	49.61	2.67, 2.02–3.53
**Women of non-white ethnic origin** 4	2001 Census, South West Wales [42]	15/398 (3.8%)	8,304/503,256 (1.65%)	9.72	2.33, 1.39–3.91
**Women smoking** 2	Infant feeding survey, pregnant women in Wales 2005 [45]	73/454 (16%)	457/2,076 (22%)	7.57	0.68, 0.52–0.89
**Women: alcohol intake, any** 2	Infant feeding survey, pregnant women in Wales 2005 [45]	193/453 (43%)	1,141/2,085 (55%)	21.44	0.61, 0.50–0.75

Notes to table: No correction taken for multiple comparisons.

1 Deprivation (Townsend) scores, ranks and fifths are based on geographical area of residence, using Lower Super Output Areas (LSOAs) defined by postcodes. This measure of material deprivation is calculated from rates of unemployment, vehicle ownership, home ownership, and overcrowding [49].

2 In five cases, both parents were students, and ONS categories could not be allocated. Fathers’ occupations taken where no occupation for mother [44], [49].

3 as reported by mothers at recruitment at 36 weeks’ pregnancy.

4 as in hospital records.

5 unequal sample sizes, unequal variances.

**Figure 5:**
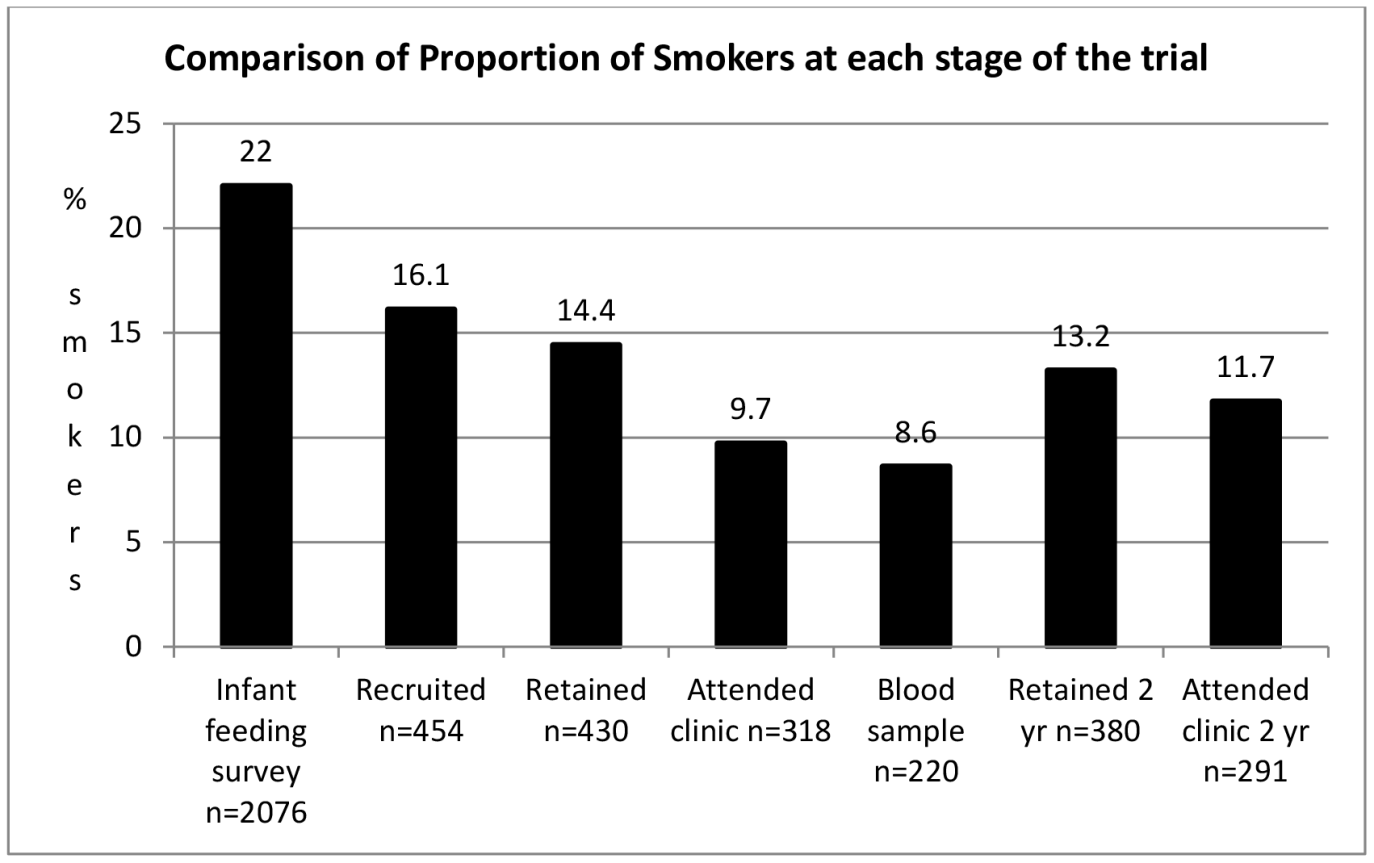
Comparison of proportion of smokers at each stage of the trial.

Census data [42] indicated that ethnic minorities were not under-represented. We recruited relatively few lone parents (19, 4.2%), when compared with households containing children of all ages in South West Wales (7.53%). No-one was classified as ‘homeless’ at recruitment. Comparisons with pregnant women in Wales suggest that the recruited sample may over-represent non-smokers (Figure 5) and alcohol abstainers [45] (Table 2).

Most, 69% (286/417 responding to the question) participants reported first hearing about the trial when they were approached by researchers in antenatal clinics. This personal approach was crucial to the decision to enrol for most participants (233/404 responding, 58%). The hope of preventing asthma or eczema in their infant was parents’ most frequently cited reason for joining, followed by interest in eczema, asthma or allergy (Table S2). Altruistic motives were also apparent: 47% (190/403) stated that helping research and 42% (166/398) that helping children were important motivators, as illustrated:

*Anything to help prevent the children of the next generation developing allergies. (Participant 147, full participation).*

But these considerations could be over-ridden:

*I wanted to help find a cure for eczema, but my family said we were being used as ‘guinea pigs’, so I stopped. (participant 118, telephone follow up).*

Of reasons for joining the trial considered (Table S2), only ‘interest in probiotics’ was associated with occupational group (χ^2^ 8.55, p = 0.003, df = 1) or deprivation (Townsend) fifth (χ^2^ 4.27, p = 0.04, df = 1).

#### b) Retention

**Figure 6:**
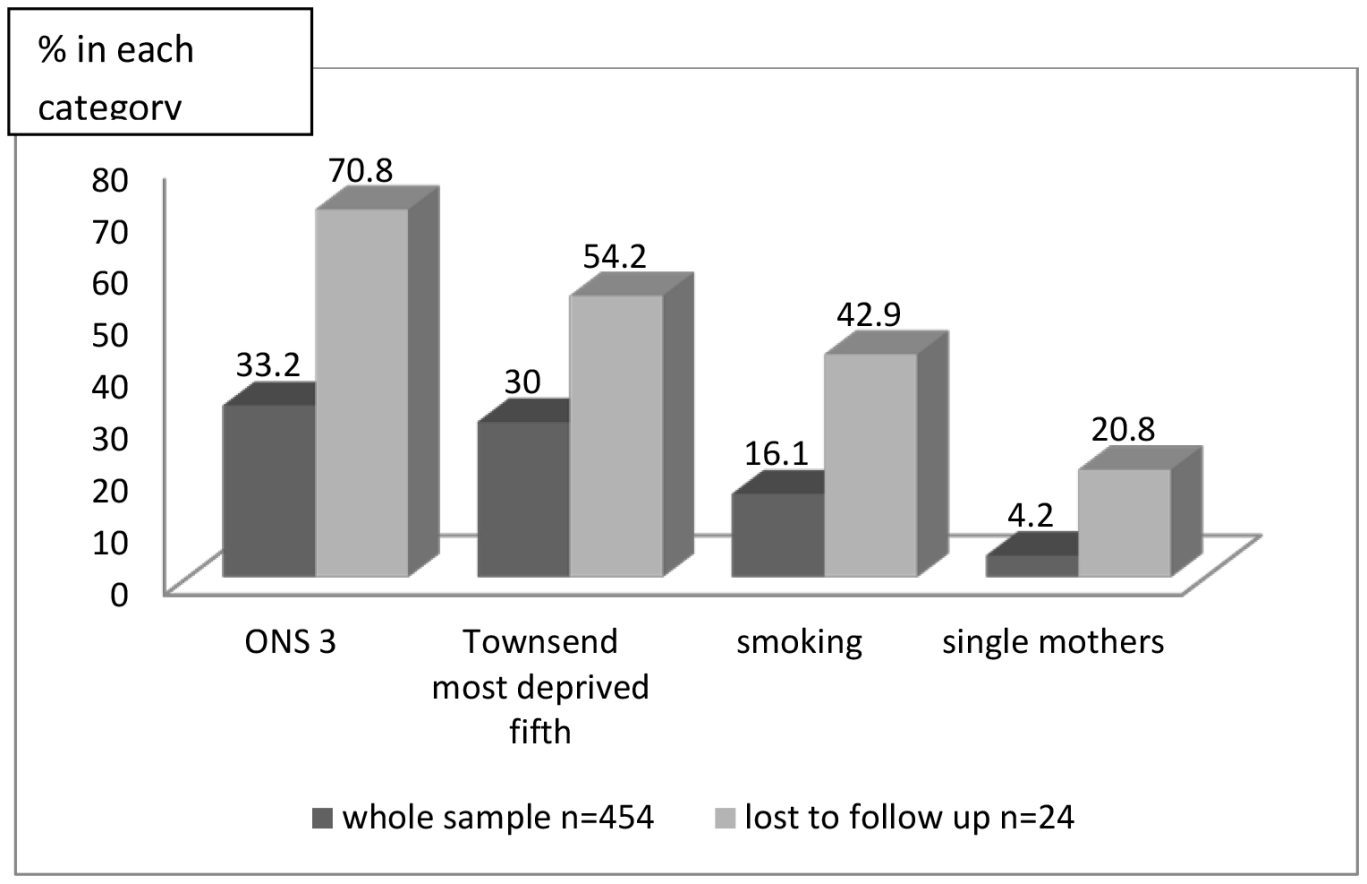
Women lost to follow up at 6 months compared with the whole sample.

Internal comparisons indicated that the most disadvantaged were less likely to be retained at 6 months. Comparisons using ONS categories and deprivation (Townsend) fifths gave similar findings: 17/24 (70.8%) lost came from ONS category 3 (routine occupations and unemployed) compared with 149 of 449 (33.2%) recruited (χ^2^ 14.46 df = 1, OR 0.19, 0.08–0.46, p<0.001); 13/24 (54.2%) were from the most materially deprived fifth compared with 123/454 (27.1%) recruited (χ^2^ 8.01, df = 1, OR 0.29, 0.13–0.67, p = 0.01) (Figure 6, Table S1).

Attrition was higher amongst smokers (χ^2^ 14.38, df = 1, OR 0.20, 0.09–0.46, p<0.001). Five of the 19 (26.3%) single mothers were lost to follow up. At 2 years, in multivariate analysis, retention at 2 years was associated with maternal age, not smoking at recruitment, occupation category, carers’ reports of rashes and reporting any adverse events during supplementation (Table 3).

**Table 3:** Factors affecting trial participation at 6 months and 2 years: adjusted analyses.

	Clinic attendance 6 months		Consent to skin-prick testing 6 months^3^	Retention in study 2 years	Attendance at clinic 2 years	Consent to skin-prick testing 2 years	Consent to blood sample donation 6 months
Numbers in analysis	413		408	408	408	408	404
	OR. (95% CI.)		OR. (95% CI.)	OR. (95% CI.)	OR. (95% CI.)	OR. (95% CI.)	OR. (95% CI.)
**Mother’s age, each year**	1.08 (1.03–1.14)		1.06 (1.01–1.11)	1.09 (1.02–1.15)	1.06 (1.02–1.10)	1.05 (1.00–1.09)	NS
**Smoking at recruitment**	0.30 (0.15–0.62)		0.32 (0.16–0.65)	0.50 (0.23–1.09)	NS	NS	0.43 (0.22–0.83)
**Deprivation**							
Most deprived fifth	0.86 (0.73–0.99)		NS	NS	0.86 (0.75–0.97)	NS	NS
**Occupation of mother**							
• ONS category 3	NS		0.75(0.57–0.99)	0.68 (0.48–0.96)	NS	0.79 (0.62–0.996)	0.72 (0.57–0.92)
**Adverse Event** 1 **to infant by 6 months**	2.36 (1.26–4.41)		1.93 (1.09–3.42)	1.95 (0.92–4.11)	1.97 (1.19–3.26)	1.78 (1.12–2.82)	NS
**Report of rash** 2	1.93 (1.03–3.60)		2.11 (1.16–3.86)	2.27 (1.14–4.35)	2.54 (1.62–4.00)	1.96 (1.27–3.02)	NS
**Interest in probiotics prompted recruitment**							
Yes, very much or to some extent*							
• Uncertain, don’t know	0.37 (0.15–0.91)		0.37 (0.17–0.86)	NS	0.50 (0.27–0.95)	0.55 (0.31–1.00)	0.28 (0.14–0.54)
• Not at all	0.25 (0.12–0.53)		0.34 (0.17–0.68)	NS	0.55 (0.32–0.94)	0.51 (0.31–0.85)	0.49 (0.29–0.82)
**Joined to help research**							
Yes, very much*							
• Yes, to some extent	0.46 (0.24–0.86)		0.42 (0.23–0.77)	NS	NS	NS	NS
• Uncertain, don’t know	0.39 (0.15–1.00)		0.35 (0.14–0.86)	NS	NS	NS	NS
• Not at all	0.23 (0.09–0.57)		0.17 (0.07–0.41)	NS	NS	NS	NS
	**Clinic attendance 6 months**		**Consent to skin-prick testing 6 months** 3	**Retention in study at 2 years**	**Attendance at clinic 2 years**	**Consent to skin-prick testing at 2 years**	**Consent to blood sample donation at 6 months**
**Joined to help children**							
Yes, very much *							
• Yes, to some extent		NS	NS	NS	NS	NS	0.50 (0.30–0.83)
• Uncertain, don’t know		NS	NS	NS	NS	NS	0.96 (0.47–2.06)
• Not at all		NS	NS	NS	NS	NS	0.29 (0.14–0.62)
**Asthma as adult, mother** NS			NS	NS	NS	NS	2.83 (1.37–5.84)
**Mother taking corticosteroids at recruitment**		NS	NS	NS	NS	NS	0.46 (0.19–1.09)
**Asthma as adult, father**		NS	NS	NS	NS	NS	1.72 (0.97–3.03)
**Trial arm: intervention**		NS	NS	NS	NS	NS	1.74 (1.13–2.69)
Hosmer and Lemeshow test (df 8)		*x* ^2^ 9.37, p 0.31	*x* ^2^ 7.11, p 0.53	*x* ^2^ 6.87, p 0.55	*x* ^2^ 1.30, p 0.99	*x* ^2^ 5.30, p 0.73	*x* ^2^ 10.71, p 0.21
Nagelkerke R^2^		0.33	*0.31*	*0.17*	*0.18*	*0.14*	*0.21*
−2 log likelihood (LL) (df)		337.24 (10)	361.33 (10)	250.41 (5)	457.704 (6)	496.332 (5)	487.47 (11)
**Predictions (% correct):**							
Overall		80.6	79.2	88.0	72.2	68.6	66.3
Participation		93.6	93.0	99.2	91.2	85.8	75
Non-participation		39.4	38.8	0	30.2	40.3	56.4

Notes to table:

Variables entered are listed in Tables S1 and S2. Categories were collapsed to avoid low numbers, as necessary. A backwards likelihood ratio criterion was used to select predictor variables. Reports of asthma and eczema were tested separately and together. Models were checked by two of us and found to be robust: removal of outliers made no overall difference. Not all participants responded to all questions. Missing data in some variables reduced the number cases in the analyses. We did not impute values.

1 Most adverse events related to symptoms typical of common problems in routine clinical practice. None were attributed to the trial intervention by the trial’s data monitoring committee [35].

2 Not all rashes had been diagnosed by a professional: carer’s report was the variable of interest.

3 Testing was not offered to 5 infants, who were excluded from this analysis. NS represents ‘not significant’. * denotes reference category.

**Clinic Attendance** and consent to skin-prick testing were necessarily closely linked. Demographic variables predicted clinic attendance and consent to testing at 6 months and 2 years (Table 3, Figures 3, 4, 5). Only the most disadvantaged categories (deprivation (Townsend) fifth and ONS category 3, routine occupations or never worked) were associated with non-attendance and declining testing. Logistic regression model parameters improved when putative motivations for clinic attendance or leverage, such as reports of rashes or adverse events, and reasons for joining the trial relating to saliency and altruism, such as ‘interest in probiotics’, were taken into consideration (Table 4). ‘Wanting to help research’, predicted involvement at 6 months, but not at 2 years (Table 3).

**Table 4:** Changes in regression models with addition of predictor variables.

	6 month clinic attendance						2 year clinic attendance						Blood sample donation				
Predictors added	Overall prediction (%)	Attenders predicted (%)	Non-attenders predicted (%)		Nagelkerke R^2^	−2 log likelihood (−2LL) (df)	Overall prediction (%)	Attenders predicted (%)	Non-attenders predicted (%)		Nagelkerke R^2^	−2 log likelihood (−2LL) (df)	Overall prediction (%)	Consent predicted (%)	Declining predicted (%)	Nagelkerke R^2^	−2 log likelihood (−2LL) (df)
i) Socio-demographic and health-related: ONS category, deprivation fifths, maternal age, smoking status, asthma or eczema in parents	75.7	91.1	38.5		0.20	469.30 (3)	67.9	86.5	32.4		0.11	541.67 (3)	62.9	72.5	53.7	0.11	576.89 (6)
ii) Leverage: reports of rash or adverse event in infant	78.2	93.7	32.7		0.22	408.74 (5)	70.9	89.7	34.2		0.17	506.96 (4)	No change				
iii) Reasons for joining the trial	80.6	92.9	40.4	0.33		337.24 (10)	72.2	91.2	30.2	0.18		457.70 (6)	66.3	75.0	56.4	0.21	487.47 (11)
**Significance of reductions in** −**2LL**																	
Addition of rashes and adverse events (leverage factors)	?^2^ 60.56, df = 2, p<0.001						?^2^ 34.67, df = 1, p<0.001						NS				
Addition of reasons for joining the trial	?^2^ 71.51, df = 5, p<0.001						?^2^ 51.22, df = 2, p<0.001						?^2^ 89.42, df = 5, p<0.001				

Note to table:

To obtain a measure of the impact of factors relating to the three categories listed, we calculated the reductions in −2LL at each stage.

**Consent to venous blood sample donation** by infants at 6 months was positively associated with professional or managerial occupations, not smoking, being in the intervention arm, interest in the trial intervention, wanting to help children and be involved in research, and experiencing asthma in adulthood. It was negatively associated with maternal use of corticosteroids (Table 3). Including factors related to reasons for joining the trial reflecting altruism, such as ‘wanting to help children’, strengthened the regression outputs. However, infants’ rashes and adverse events were not associated with consent (Table 4).

### 2) Data Weighting to Assess Volunteer Bias

Weighting the data according to the distribution of material deprivation (Townsend) fifths relative to the SAIL database (Table S5) changed some trial findings, but not others (Table 5). *Post hoc* subgroup analyses of deprivation (Townsend) fifths indicated that findings were unchanged where the impact of the intervention was concentrated in the under-represented group, the most deprived (atopic sensitisation). However, findings were modified where impact of the intervention was concentrated in the over-represented group, the least deprived (atopic eczema). The absolute risk reduction was changed by 36.2%, from 6.9% (0.9–13.1%) to 4.6% (−1.4–10.5%), and the odds ratio by 40%, from 0.40 (0.18–0.91) to 0.56 (0.26–1.21) (Table 5). Doctor-diagnosed eczema appeared to be more common in the least deprived participants, and asthma in the most deprived, but differences were not statistically significant. Interaction terms between treatment arm and material deprivation (as Townsend fifths) were not significant.

**Table 5:** Clinical outcomes by 2 years according to trial arm: weighted, unweighted and subgroup analyses.

	Unweighted analysis: Whole sample				Weighted analysis: Whole sample				Unweighted analysis: Least deprived fifth.			Unweighted analysis: Deprivation fifths 2–4 only			Unweighted analysis: Most deprived fifth		
Variable	Probiotic arm N = 220 n(%)	Placebo arm N = 234 n(%)	OR (95% CI)	ARR% (95% CI)	Probiotic arm n(%)	Placebo arm n(%)	OR (95% CI)	ARR% (95% CI)	Probiotic arm N = 66 n(%)	Placebo arm N = 70 n(%)	OR (95% CI)	Probiotic arm N = 99 n(%)	Placebo arm N = 96 n(%)	OR (95% CI)	Probiotic arm N = 55 n(%)	Placebo arm N = 68 n(%)	OR (95% CI)
**Atopic sensitisation**																	
Positive to ≥1 allergen at either 6 months or 2 years	18/171 (10·5)	32/173 (18·5)	0·52 (0·28–0·98)	8.0* (0.5–15.4)	18/169 (10.7)	32/172 (18.6)	0.52 (0.28–0.97)	8.0* (0.5–15.4)	7/58 (12.1)	12/64 (18·8)	0·60 (0·22–1·63)	9/77 (11.7)	10/71 (14·1)	0·81 (0·31–2·12)	2/36 (5.6)	10/38 (26·3)	0·17 (0·03–0·82)
**Skin conditions**																	
Atopic eczema	9/171 (5·3)	21/173 (12·1)	0·40 (0·18–0·91)	6.9* (0.9–13.1)	11/170 (6.50)	19/172 (11.0)	0.56 (0.26–1.21)	4.6* (–1.4–10.5)	2/58 (3·4)	11/64 (17·2)	0·17 (0·04–0·81)	5/77 (6.5)	5/71 (7·0)	0·91 (0·25–3·31)	2/36 (5.6)	5/38 (13.2)	0·39 (0·07–2·14)
Eczema diagnosed by a doctor	73/214 (34·1%)	72/222 (32·4%)	1·07 (0·72–1·60)	1.7† (–7.1–10.5)	75/205 (36.6)	71/219 (32.4)	1.20 (0.81–1.80)	4.2† (–4.9–13.2)	21/66 (31·8%)	28/68 (41·2%)	0·67 (0·33–1·35)	34/97 (35·1%)	26/90 (28·9%)	1·33 (0·72–2·46)	18/51 (35.3)	18/64 (28·1)	1·40 (0·63–3·08)
**Respiratory conditions**																	
Asthma diagnosed by a doctor	22/193 (11.4)	20/199 (10.1)	1.15 (0.61–2.19)	0.2† (–6.2–6.7)	24/189 (12.7)	21/200 (10.5)	1.24 (0.67–2.31)	1.1† (–5.1–7.4)	5/64 (7.8)	5/65 (7.7)	1.02 (0.28–3.70)	10/87 (11.5)	8/79 (10.1)	1.50 (0.43–3.09)	7/42 (16.7)	7/55 (12·7)	1·37 (0·44–4·27)

**Notes to table:**

ARR (absolute risk reduction), calculated only for whole sample. * favours probiotics † favours placebo.

Weighted numbers differ from original numbers. Cell counts were rounded by spss.

All sample, intention to treat.

## Discussion

Potential for volunteer bias, created at recruitment, intensified throughout the trial (Figures 3, 4, 5). Retention and participation were associated with socio-demographic variables, smoking status and variables reflecting leverage, saliency and altruism. Trial findings were modified by data weighting to account for volunteer bias (Table 5).

### Limitations and Strengths

From **single site research**, we cannot assume that respondents and response patterns are representative of other populations. Unusually for a clinical trial [51], the lead institutions are in an area of the European Union (EU) where GDP is 75% below the community average, a Convergence area [52]. Trial location may have influenced recruitment, retention, and sample donation. For example, attitudes towards blood donation differ between communities [53]–[55].

This trial was **restricted to healthy dyads**. To our knowledge, predictors of carers’ consent to blood sample donation by well infants have not been explored in other trials, and associations reported here require testing in other populations [50]. However, cohort studies report similar clinic attendance rates [56]. The balance between benefit and harm is more uncertain in prevention or vaccine trials involving healthy participants than in therapeutic trials [57]. Further work is needed to explore generalisation of these findings to trials involving unwell or hospitalised children, where recruitment is restricted to closely defined populations with current medical conditions [14], [58]–[60].

**Comparison with external data** was the only option available to evaluate demographic representation at recruitment; however, some ages, locations and time-frames were not entirely congruent. Therefore, we tested this approach by comparing the deprivation scores and rankings of respondents with those of women giving birth in the same timeframe and geographical areas. We are unaware of other trials testing sample selection using this approach. The similarity between the comparisons indicates that it would be reasonable to assess volunteer bias using Census data where closely matched population data is unavailable.

The data sources used for comparison are themselves vulnerable to social desirability, volunteer and non-response bias, and may not be fully representative of the population. For example, the 2001 Census had a 93–94% response rate in Wales, falling below 90% for women aged 20–24 [61]: the most disadvantaged are likely to be under-represented [62]. Accordingly, our calculations may underestimate demographic imbalance. Reports of behaviour are vulnerable to **social desirability response biases**, but we have no reason to assume that our data would be uniquely vulnerable.

**Non-contact bias** should be distinguished from volunteer bias [13]. The recruited sample’s composition may have been influenced by the characteristics of women attending ante-natal clinics and community groups. Marginalised women may not access care or only accept domiciliary care in refuges, so would neither have received our invitation letters nor been approached (Table 1).

**Interpretation** of weighted analyses rests with readers; this strategy to account for volunteer or non-response bias is routine in observation studies, including UK birth cohorts [25], [63]–[65]. We acknowledge the limitations of *post hoc* subgroup analyses [66], [67], and present these solely to illustrate how outcome distribution affects data weighting, not to guide clinical practice. Low numbers in outcome variables necessitate cautious interpretation; however, these findings merit exploration in pooled data sets and meta-analysis.

### 1. Potential for Volunteer Bias

- **Recruitment** strategies in this trial favoured wealthier families with healthier behaviours, as in observation studies [17]–[19], [23]–[25], cluster [26] and adult prevention trials [10], [12], [27]. Significant degrees of sub-optimal recruitment and potential volunteer bias are relatively recent phenomena [68], [69]. Just as recruitment to trials is becoming increasingly difficult [2], successive UK birth cohorts have had lower response rates. While the 1958 & 1970 MRC cohorts recruited 98.76% & 95.86% (17416/17634 & 16571/17287) of those approached [70], [71], the Millenium Cohort had a 68% unweighted response rate (72% in Wales) [25].
- **Retention** was influenced by socio-demographic and less tangible factors.
  - The most disadvantaged and smokers were less likely to participate in follow-up, attend clinics, consent to skin-prick testing or blood sample donation. Treatment allocation had no negative impact.
  - ii) Potential for volunteer bias in the retained samples was not confined to socio-demographic parameters [72], [73]. Multivariate analyses indicated that when demographics were accounted, leverage, saliency [13], [74] and altruism [14], [75] are important predictors of participation (Table 4). To our knowledge, this has not been tested in trial data.

The **saliency and leverage** of the trial, clinic or skin-prick testing, and the theory of social exchange [74], [76], [77] featured in binary, threshold decisions to participate. Opportunities to see consultant paediatricians and receive allergen testing may have been particularly attractive to carers of infants experiencing adverse events or rashes. Access to treatment [22] or expectation of better attention incentivise participation [59], [78].

**Altruism** was important in the decision to consent to venous blood sample donation by well infants. Here, there were no possible direct benefits to the family, and the infant’s discomfort was a deterrent [79]. Leverage related to clinic attendance and skin-prick testing was discounted, and ‘wanting to help children’ predicted consent. Requests for time and biological samples deter many potential trial participants [1], [17], [30], [80], [81]. However, 220 participants consented to sample donation. Such altruism is more evident in less recent trials [75].

### 2. Volunteer Bias in Trials and Data Weighting

Applying the concept of volunteer bias to trial data tests the generalisability, external validity, transferability, utility and dependability of trial findings. Keyword searches in three databases (PubMed, Web of Science, Scopus) indicate that data weighting to account for and quantify potential volunteer bias is rarely undertaken in paediatric prevention trials.

### Generalising the Findings

Findings (Table 5) suggest that to minimise any risk that results may be distorted by systematic differences between participants and the population likely to use the trial’s findings, outcomes should be assessed in samples as free of volunteer bias as possible [7], [26]. Although an unrepresentative sample does not necessarily mean that findings would not be replicated in a wider population, research quality criteria include non-biased sample selection [82]. This is particularly important where participants’ characteristics influence study outcomes [8], [13], [19], [32], [83]. Strategies to account for missing data, such as sensitivity analysis, do not address volunteer bias [84]. It cannot be assumed that participation and attrition are random events, prompting calls for full details of target or eligible populations to be reported for all trials [20].

### Power of the Trial: Recruiting the Target Population

Problems were confined to the most materially disadvantaged and smokers. Non-targeted recruitment and retention risk volunteer bias and disenfranchisement of the least affluent and most marginalised, where childhood ill-health is concentrated [85]. Many outcomes in health services’ research, including childhood asthma and wheezing, are affected by material deprivation [16], [85], [86], or geographical location [87]. Eczema is associated with urbanisation [88] and parents’ educational attainment [89], both linked with reduced deprivation. Here, doctor-diagnosed eczema was no less common in the over-represented group (the affluent) (Table 5), indicating that volunteer bias did not reduce the study’s power for this outcome. However, asthma was less common in the over-represented group. For this outcome, it will be important to consider any potential loss of power, as the event rate proportion may differ between the population and the recruited and retained samples.

### Robust Trial Findings: Suggestions and Solutions

Weighting increased the leverage of data from the most deprived participants (Table 5). Accordingly, this confirmed the robustness of positive outcomes concentrated in under-represented groups (atopic sensitisation). However, where the intervention’s impact was concentrated in over-represented groups (atopic eczema), weighting changed both the absolute and relative effects of the intervention. Weighting techniques, standard practice in cohort studies [25], [63]–[65], based on demographic distribution at recruitment, can augment analyses of trial data [8], [41]. Such weighting is based on assumptions that participants from disadvantaged groups are representative or typical of their groups in all respects, including attributes not recorded; only careful fieldwork and local knowledge can support such suppositions. Obviating any need for such subjective judgments, and obtaining trial evidence on which to base practice recommendations to the wider, target population, necessitates engagement, recruitment and retention of fully representative samples [34], [82]. Strategies include:

- Additional resources. Trialists are under pressure to recruit to safeguard their sponsors’ investments. However, the disadvantaged are disproportionately hard to reach [13], [21]. To safeguard investment in clinical trials, the research community should budget sufficient time and resources for complicated, personalised contact and follow up procedures [18], [28], as in birth cohorts [25], [63].
- Stratification of the population and over-sampling those least likely to participate, as in cohort studies [25], [63]–[65].
- Electronic follow up using routinely collected health services’ data, where available. More work is needed to evaluate this approach and assess the traceability of respondents.
- Weighted analysis to account for residual problems. Accounting for all possible confounders will be difficult, but even partial mapping strengthens the analysis [41].

### Conclusions

If trial evidence is to reflect population diversity, demographically representative samples should be recruited and retained. Disproportionate socio-demographic representation arising at recruitment intensified throughout the trial. Accounting for this by data weighting to assess volunteer bias modified important trial findings. Whether this would occur in other trials warrants investigation. However, material deprivation is not the only predictor of participation. The leverage-saliency theory of research participation remains important; additionally, these findings indicate that altruism should not be discounted. Application of the concept of volunteer bias to clinical trials suggests that to offer reassurance regarding the generalisability, external validity, transferability, utility and dependability of findings, researchers should quantify differences between recruited samples and target populations and weight data to protect findings from potential distortion by volunteer bias.

## Supporting Information

Table S1 Variables entered into regression models, whole sample and sample retained at 6 months.
(DOC)

Table S2 Reasons for joining the trial (n = 430).
(DOC)

Table S3 Occupational groups in recruited sample and 2001 Census for South West Wales: mothers**.**
(DOC)

Table S4 Occupational groups in recruited sample and 2001 Census for South West Wales: fathers**.**
(DOC)

Table S5 Proportions used for Data Weighting for each outcome by Deprivation (Townsend) Quintile.
(DOC)
