# Supplementary material for: Volunteer Bias in Recruitment, Retention, and Blood Sample Donation in a Randomised Controlled Trial Involving Mothers and Their Children at Six Months and Two Years: A Longitudinal Analysis
Source: PLoS One. 2013 Jul 9;8(7):e67912. doi: 10.1371/journal.pone.0067912 (PMC3706448; doi:10.1371/journal.pone.0067912)
Supplement: Table S1 — Variables entered into regression models, whole sample and sample retained at 6 months. (DOC) [file pone.0067912.s001.doc]

**Table S1. Variables entered into regression models, whole sample and sample retained at 6 months**

| **Interval variables** | Whole sample (n=454)  (mean [SD], median, inter-quartile and full range) | Retained in the study at 6 months (n=430)  (mean [SD], median, inter-quartile and full range) | |
| --- | --- | --- | --- |
| Mother’s age at delivery | 29.25 (5.4), 30, 25-33.75, 17-44 | 29.4 (5.74), 30, 25-34, 17-44 | |
| Deprivation score^1^ | 0.09 [4.21], -0.40, -3.43-+3.26, -7.50-+10.40 | -0.068 [4.17], -0.775, -3.43-+3.11,-7.50-+10.40 | |
| Deprivation rank^1^ | 925.58 [624.1], 926, 329-1555, 1-1894 | 902.21 [620.41], 855.50, 329-1533, 1-1894 | |
| **Categorical variables** |  | Whole sample (n=454) | Retained in study (n=430) |
| Trial arm | Allocated supplement | 220 | 208 |
|  | Allocated placebo | 234 | 222 |
| Mother with asthma^2^ as adult | Yes | 110 | 101 |
|  | No | 344 (75.8%) | 329 (76.5%) |
| Mother with eczema^2^ as adult | Yes | 88 | 84 |
|  | No | 365 (79.7%) | 345 (80.4%) |
| Mother with asthma^2^ or eczema^2^ as adult | Yes | 156 | 145 |
|  | No | 298 (65.6%) | 285 (66.3%) |
| Mother taking corticosteroids | Yes | 60 | 58 |
|  | No | 394 (86.8%) | 372 (88.5%) |
| Father with asthma^2^ as adult | Yes | 83 | 79 |
|  | No | 371 (81.7%) | 351 (82.6%) |
| Father with eczema^2^ as adult | Yes | 50 | 48 |
|  | No | 404 (99.1%) | 382 (88.8%) |
| Father with asthma^2^ or eczema^2^ as adult | Yes | 122 | 116 |
|  | No | 332 (73.1%) | 314 (73.0%) |
| Siblings with asthma^2^ | 1 or more | 55 | 50 |
|  | None | 398 (86.1%) | 380 (88.4%) |
| Siblings with eczema^2^ | 1 or more | 111 | 107 |
|  | None | 343 (75.6%) | 323 (75.1%) |
| Siblings with asthma^2^ or eczema^2^ | 1 or more | 131 | 124 |
|  | None | 323(71.1%) | 306 (71.2%) |
| Smoking at recruitment | Yes | 73 (16.1%) | 62 (14.4%) |
| Self-reported | No | 381 | 368 |

| **Categorical variables** |  | Whole sample (n=454) | Retained in study (n=430) |
| --- | --- | --- | --- |
| Deprivation^1^ fifth | Least deprived 20% | 136 (30.0%) | 135 (31.4%) |
|  | 2^nd^ least deprived 20% | 65 (14.3%) | 61 (14.2%) |
|  | Middle fifth (40-60%) | 79 (17.4%) | 76 (17.7%) |
|  | 2^nd^ most deprived 20% | 51 (11.2%) | 48 (11.2%) |
|  | Most deprived 20% | 123 (27.1%) | 110 (25.6%) |
| Occupation of mother. ONS categories^2^ | Managerial and professional. ONS 1. | 167 (37.2%) | 163 (38.4%) |
|  | Intermediate ONS 2 | 133 (29.6%) | 130 (30.6%) |
|  | Routine occupations and never worked ONS 3 | 149 (33.2%) | 132 (31.1%) |
| Alcohol in pregnancy | None | 258 (57.2%) | 242 (56.7%) |
|  | 1 unit per week or less | 187 (41.4%) | 180 (42.1%) |
|  | >1 unit/ week | 6 | 5 |
| Rash reported by carer at | No | NA | 297 |
| 6 months (n=428) | Yes |  | 131 (30.6%) |
| Adverse events reported by 6 months | No | 305 | 289 |
|  | Yes | 148 (32.8%) | 141 (32.8%) |
| Rash reported by 2 years (n=440) | No | NA | 189 (43%) |
|  | Yes |  | 251 (57%) |
| Recruited in person | Yes | 418 (92.1%) | 394 (91.6%) |
| Recruited by post | No | 36 | 36 |
| Comments on trial | Positive |  | 169 (40%) |
| (n=422) | None or neutral |  | 241 (57.1%) |
|  | Negative |  | 12 (2.8%) |

**Notes to table** SD represents standard deviation

^1^Respondents’ postcodes at birth were mapped to the Lower Super Output Area (LSOA) codes using the "Postcode Directory" in UKBORDERS [1]. LSOA codes were mapped to the Townsend Index of Deprivation, using a file provided by Health Solutions Wales to yield deprivation (Townsend) scores, ranks, fifths and deciles within Wales for the LSOA codes of residence. Ranks in Wales range 1-1896.

^2^Condition diagnosed by a professional, as reported at recruitment

^3^The mother’s category was used unless she was a student or not working long-term [44]. Several mothers who were not working lived in non-deprived areas, and the codes of their economically dominant partners better reflected their lifestyles or ‘household class’ [49:351].

Smoking, maternal age and deprivation (Townsend) score were all associated with occupational group (linear by linear χ^2^ 32.9, df 1, p<0.001; Kruskal Wallis χ^2^ 51.64, df 2, p<0.001; Kruskal Wallis χ^2^ 83.6, df 2, p<0.001); smoking was associated with maternal age and deprivation score (U 8631.5, z -4.98 p<0.001, U 7561.5, z -6.18, p<0.001).

[1] UK Borders (2009) Postcode directory resources**.** Available: <http://edina.ac.uk/ukborders/>. Accessed 4 December 2012.
