# Supplementary material for: Volunteer Bias in Recruitment, Retention, and Blood Sample Donation in a Randomised Controlled Trial Involving Mothers and Their Children at Six Months and Two Years: A Longitudinal Analysis
Source: PLoS One. 2013 Jul 9;8(7):e67912. doi: 10.1371/journal.pone.0067912 (PMC3706448; doi:10.1371/journal.pone.0067912)
Supplement: Table S2 — Reasons for joining the trial (n = 430). (DOC) [file pone.0067912.s002.doc]

**Table S2 Reasons for joining the trial (n=430)**

|  | **Very positive influence**  **n (% responding)** | **Positive influence to some extent**  **n (% responding)** | **Uncertain influence, don’t know n (% responding)** | **Not at all influential**  **n (% responding)** | **Negative influence n (% responding)** | **No answer**  **n (% sample)** |
| --- | --- | --- | --- | --- | --- | --- |
| **Personal approach by researcher*** | 233/404 (58) | 101/404 (25) | 20 /404 (5) | 50/404 (12) | 0 | 26/430 (6) |
| **Written information** | 101/400 (25) | 176/400 (44) | 35/400 (9) | 83/400 (21) | 5/400 (1) | 30/430 (7) |
| **Healthcare professionals** | 35/395 (9) | 52/395 (13) | 58/395 (15) | 244/395 (62) | 6/395 (1.5) | 35/430 (8) |
| **Media** | 14/401 (4) | 32/401 (8) | 61/401 (15) | 286/401 (71) | 8/401 (2) | 29/430 (7) |
| **Preventing eczema and asthma in my child** | 351/414 (85) | 35/414 (9) | 12/414 (3) | 14/414 (3) | 2/414 (0.5) | 16/430 (4) |
| **Interest in eczema, asthma and allergy** | 315/413 (76) | 48/413 (12) | 15/413 (4) | 33/413 (8) | 2/413 (0.5) | 17/430 (4) |
| **Interest in probiotics** | 58/413 (14) | 84/413 (20) | 89/413 (21) | 181/413 (44) | 1/413 (0.2) | 17/430 (4) |
| **Wanting to help children** | 166/414 (40) | 141/414 (34) | 58/414 (14) | 48/414 (12) | 1/414 (0.2) | 16/430 (4) |
| **Wanting to help research** | 190/413 (46) | 146/413 (35) | 40/413 (10) | 36/413 (9) | 1/413 (0.2) | 17/430 (4) |
| **Support of family** | 152/413 (37) | 119/413 (29) | 62/413 (15) | 75/413 (18) | 5/413 (1.2) | 17/430 (4) |

**Note to table**

Not all participants responded to all questions.
