# Supplementary material for: Volunteer Bias in Recruitment, Retention, and Blood Sample Donation in a Randomised Controlled Trial Involving Mothers and Their Children at Six Months and Two Years: A Longitudinal Analysis
Source: PLoS One. 2013 Jul 9;8(7):e67912. doi: 10.1371/journal.pone.0067912 (PMC3706448; doi:10.1371/journal.pone.0067912)
Supplement: Table S3 — Occupational groups in recruited sample and 2001 Census for South West Wales: mothers. (DOC) [file pone.0067912.s003.doc]

**Table S3: Occupational groups in recruited sample and 2001 Census for South West Wales: mothers**

Occupational Group Sample 2001 Census for SW Wales

Number (% in sample) Number (% for Census)

1: Managers, Senior Officials 38 (8.37%) 7,532 (7.68%)

2: Professionals 63 (13.88%) 8,338 (8.50%)

3: Associate Professionals, 87 (19.16%) 12,489 (12.73%)

Technical Occupations

4: Admin or Secretarial 74 (16.30%) 19,618 (20.00%)

5: Skilled Trades 7 (1.54%) 1,979 (2.02%)

6: Personal Services 54 (11.89%) 12,352 (12.59%)

7: Sales & Customer Services 64 (14.10%) 13,094 (13.34%)

8: Machine, Plant Operatives 9 (1.98%) 3,638 (3.71%)

9: Elementary Occupations 23 (5.07%) 12,581 (12.82%)

10: Never Worked 9 (1.98%) 388 (0.40%)

11: Not Worked For 2 Years 9 (1.98%) 1,421 (1.45%)

Or More

12: Full-time Students 17 (3.74%) 4,706 (4.80%)

Total 454 (100%) 98,136 (100%)

**Notes to table**

This table summarises the distributions across occupational groups in our sample and the corresponding section of the population, based on the 2001 Census for SW Wales [42]. Census figures include women aged 16-74, and our respondents were aged 17-44.

Categories absent from our data include ‘Retired’, ‘Part-time Student’, ‘Long-term Sick and Disabled’, ‘Looking After Family’, ‘Currently Not Working’, ‘Other’.

The chi-squared statistic for the whole table is 89.35, df=11, p<0.001; chi-squared for linear-by-linear association is 18.90, df=1, p<0.001.
