# Supplementary material for: Volunteer Bias in Recruitment, Retention, and Blood Sample Donation in a Randomised Controlled Trial Involving Mothers and Their Children at Six Months and Two Years: A Longitudinal Analysis
Source: PLoS One. 2013 Jul 9;8(7):e67912. doi: 10.1371/journal.pone.0067912 (PMC3706448; doi:10.1371/journal.pone.0067912)
Supplement: Table S4 — Occupational groups in recruited sample and 2001 Census for South West Wales: fathers. (DOC) [file pone.0067912.s004.doc]

**Table S4: Occupational groups in recruited sample and 2001 Census for South West Wales: fathers**

Occupational Group Sample 2001 Census for SW Wales

Number (% in sample) Number (% for Census)

1: Managers, Senior Officials 58 (13.18%) 14,772 (12.97%)

2: Professionals 67 (15.23%) 11,647 (10.23%)

3: Associate Professionals, 64 (14.55%) 13,599 (11.94%)

Technical Occupations

4: Admin or Secretarial 15 (3.41%) 5,695 (5.00%)

5: Skilled Trades 106 (24.09%) 22,366 (19.64%)

6: Personal Services 7 (1.59%) 2,603 (2.29%)

7: Sales & Customer Services 22 (5.00%) 4,469 (3.92%)

8: Machine, Plant Operatives 50 (11.36%) 17,439 (15.32%)

9: Elementary Occupations 39 (8.86%) 14,058 (12.35%)

10: Never Worked 3 (0.68%) 817 (0.72%)

11: Not Worked For 2 Years 2 (0.46%) 2,682 (2.36%)

Or More

12: Full-time Students 7 (1.59%) 3,713 (3.26%)

Total 440 (100%) 113,860 (100%)

**Notes to table**

This table summarises the distributions across occupational groups in our sample and the corresponding section of the population, based on the 2001 Census for SW Wales [42]. Categories absent from our data include ‘Retired’, ‘Part-time Student’, ‘Long-term Sick and Disabled’, ‘Looking After Family’, ‘Currently Not Working’, ‘Other’.

In 14 cases, the mother was unable to give information about the father, including occupation.

The chi-squared statistic for the whole table is 41.33, df=11, p<0.001; chi-squared for linear-by-linear association is 20.61, df=1, p<0.001.
