# Supplementary material for: Volunteer Bias in Recruitment, Retention, and Blood Sample Donation in a Randomised Controlled Trial Involving Mothers and Their Children at Six Months and Two Years: A Longitudinal Analysis
Source: PLoS One. 2013 Jul 9;8(7):e67912. doi: 10.1371/journal.pone.0067912 (PMC3706448; doi:10.1371/journal.pone.0067912)
Supplement: Table S5 — Proportions used for Data Weighting for each outcome by Deprivation (Townsend) Quintile. (DOC) [file pone.0067912.s005.doc]

**Table S5. Proportions used for Data Weighting for each outcome by Deprivation (Townsend) Quintile**

| **Atopic Sensitisation** | | | | | |
| --- | --- | --- | --- | --- | --- |
|  | | | | | |
| Deprivation (Townsend) quintile | Population % | Sample % | Weighting factors |  | |
|  |  |  |  |  | |
| Least deprived 1 | 19.25 | 35.5 | 0.5423 |  | |
| 2 | 17.63 | 15.4 | 1.1448 |  | |
| 3 | 17.54 | 16.6 | 1.0566 |  | |
| 4 | 16.48 | 11 | 1.4982 |  | |
| Most deprived 5 | 29.1 | 21.5 | 1.3535 |  | |
|  | 100 | 100 |  |  | |
| **Atopic eczema** | | | | | |
|  | | | | | |
| Deprivation (Townsend) quintile | Population % | Sample % | Weighting factors | |  |
|  |  |  | |  | |
| Least deprived 1 | 19.25 | 35.5 | 0.5423 |  | |
| 2 | 17.63 | 15.4 | 1.1448 |  | |
| 3 | 17.54 | 16.6 | 1.0566 |  | |
| 4 | 16.48 | 11 | 1.4982 |  | |
| Most deprived 5 | 29.1 | 21.5 | 1.3535 |  | |
|  | 100 | 100 |  |  | |
| **Eczema diagnosed by a doctor** | | | | | |
|  | | | | | |
| Deprivation (Townsend) quintile | Population % | Sample % | Weighting factors | |  |
|  |  |  | | | |
| Least deprived 1 | 19.25 | 30.7 | 0.627 |  | |
| 2 | 17.63 | 14 | 1.2593 |  | |
| 3 | 17.54 | 17.7 | 0.991 |  | |
| 4 | 16.48 | 11.2 | 1.4714 |  | |
| Most deprived 5 | 29.1 | 26.4 | 1.1023 |  | |
|  | 100 | 100 |  |  | |
| **Asthma diagnosed by a doctor** | | | | | |
|  | | | | | |
| Deprivation (Townsend) quintile | Population % | Sample % | Weighting factors | |  |
|  |  |  |  |  | |
| Least deprived 1 | 19.25 | 32.9 | 0.5851 | |  |
| 2 | 17.63 | 13.8 | 1.2775 | |  |
| 3 | 17.54 | 17.3 | 1.0139 | |  |
| 4 | 16.48 | 11.2 | 1.4714 | |  |
| Most deprived 5 | 29.1 | 24.7 | 1.1781 | |  |
|  | 100 | 99.9 |  | |  |

Weighting factors were calculated in excel, and entered into SPSS syntax.

STROBE Statement—Checklist of items that should be included in reports of ***cohort studies***

|  | Item No | Recommendation | where |
| --- | --- | --- | --- |
| **Title and abstract** | 1 | (*a*) Indicate the study’s design with a commonly used term in the title or the abstract | Title |
|  |  | (*b*) Provide in the abstract an informative and balanced summary of what was done and what was found | Abstract |
| Introduction | | |  |
| Background/rationale | 2 | Explain the scientific background and rationale for the investigation being reported | p.4-5 |
| Objectives | 3 | State specific objectives, including any prespecified hypotheses | p.5 last para |
| Methods | | |  |
| Study design | 4 | Present key elements of study design early in the paper | p.5 last para, p.6 para 1 |
| Setting | 5 | Describe the setting, locations, and relevant dates, including periods of recruitment, exposure, follow-up, and data collection | p.6-7 |
| Participants | 6 | (*a*) Give the eligibility criteria, and the sources and methods of selection of participants. Describe methods of follow-up | p.6, para 1 & 3 |
|  |  | (*b*) For matched studies, give matching criteria and number of exposed and unexposed | NA |
| Variables | 7 | Clearly define all outcomes, exposures, predictors, potential confounders, and effect modifiers. Give diagnostic criteria, if applicable | Outcomes: Study objectives p.5 last para. Trial outcomes: table 5. Predictors: Appendix tables 1,2 |
| Data sources/ measurement | 8* | For each variable of interest, give sources of data and details of methods of assessment (measurement). Describe comparability of assessment methods if there is more than one group | *p.7 para 2* |
| Bias | 9 | Describe any efforts to address potential sources of bias | Multivariate analyses, by SJ & AW p.8 analysis |
| Study size | 10 | Explain how the study size was arrived at | p.6 para 2 sample size |
| Quantitative variables | 11 | Explain how quantitative variables were handled in the analyses. If applicable, describe which groupings were chosen and why | Analysis p.8. Also notes to tables 2-4 |
| Statistical methods | 12 | (*a*) Describe all statistical methods, including those used to control for confounding | Analysis p.8 |
|  |  | (*b*) Describe any methods used to examine subgroups and interactions | Analysis p.8 |
|  |  | (*c*) Explain how missing data were addressed | No adjustments were made. |
|  |  | (*d*) If applicable, explain how loss to follow-up was addressed | No adjustments. This was a study outcome. |
|  |  | (*e*) Describe any sensitivity analyses | NA |
| Results | | |  |
| Participants | 13* | (a) Report numbers of individuals at each stage of study—eg numbers potentially eligible, examined for eligibility, confirmed eligible, included in the study, completing follow-up, and analysed | Figs 1 and 2, Table 3 |
|  |  | (b) Give reasons for non-participation at each stage | Figs 1 & 2 |
|  |  | (c) Consider use of a flow diagram | Figs 1 & 2 |
| Descriptive data | 14* | (a) Give characteristics of study participants (eg demographic, clinical, social) and information on exposures and potential confounders | Appendix tables 1, 5,  Figs 3-6 |
|  |  | (b) Indicate number of participants with missing data for each variable of interest | Appendix tables 1,2 |
|  |  | (c) Summarise follow-up time (eg, average and total amount) | Figs 1,2, Table 3 |
| Outcome data | 15* | Report numbers of outcome events or summary measures over time | Tables 3-5, figs 3-6. |
| Main results | 16 | (*a*) Give unadjusted estimates and, if applicable, confounder-adjusted estimates and their precision (eg, 95% confidence interval). Make clear which confounders were adjusted for and why they were included | Unadjusted analyses p.9 para 1, Tables 2,5, appendix tables 3-4. Adjusted analyses, table 3,4. Potential confounders appendix tables 1,2. |
|  |  | (*b*) Report category boundaries when continuous variables were categorized | Appendix table 1 |
|  |  | (*c*) If relevant, consider translating estimates of relative risk into absolute risk for a meaningful time period | Table 5 |
| Other analyses | 17 | Report other analyses done—eg analyses of subgroups and interactions, and sensitivity analyses | p.11 para 2 |
| Discussion | | |  |
| Key results | 18 | Summarise key results with reference to study objectives | Discussion para 1 p.11 para 3 |
| Limitations | 19 | Discuss limitations of the study, taking into account sources of potential bias or imprecision. Discuss both direction and magnitude of any potential bias | p.11-13 |
| Interpretation | 20 | Give a cautious overall interpretation of results considering objectives, limitations, multiplicity of analyses, results from similar studies, and other relevant evidence | p.13 |
| Generalisability | 21 | Discuss the generalisability (external validity) of the study results | p.14-15 |
| Other information | | |  |
| Funding | 22 | Give the source of funding and the role of the funders for the present study and, if applicable, for the original study on which the present article is based | Acknowledgements |

*Give information separately for exposed and unexposed groups.
